# The Subtle Balance between Lipolysis and Lipogenesis: A Critical Point in Metabolic Homeostasis

**DOI:** 10.3390/nu7115475

**Published:** 2015-11-13

**Authors:** Chiara Saponaro, Melania Gaggini, Fabrizia Carli, Amalia Gastaldelli

**Affiliations:** 1 Cardiometabolic Risk Unit, Institute of Clinical Physiology, CNR, via Moruzzi, 1 56124 Pisa, Italy; chiara.saponaro@gmail.com or csaponaro@ifc.cnr.it (C.S.); melania.gaggini@alice.it or mgaggini@ifc.cnr.it (M.G.); fabrycarli@hotmail.it or fcarli@ifc.cnr.it (F.C.); 2 Dipartimento di Biotecnologie, Chimica e Farmacia, Università di Siena, 53100 Siena, Italy; 3 Dipartimento di Patologia Chirurgica, Molecolare Medica e di Area Critica, Università di Pisa, 56126 Pisa, Italy

**Keywords:** lipotoxicity, lipolysis, *de novo* lipogenesis, glyceroneogenesis, fatty liver, NAFLD, ectopic fat, HCC, SCD-1, saturated fat

## Abstract

Excessive accumulation of lipids can lead to lipotoxicity, cell dysfunction and alteration in metabolic pathways, both in adipose tissue and peripheral organs, like liver, heart, pancreas and muscle. This is now a recognized risk factor for the development of metabolic disorders, such as obesity, diabetes, fatty liver disease (NAFLD), cardiovascular diseases (CVD) and hepatocellular carcinoma (HCC). The causes for lipotoxicity are not only a high fat diet but also excessive lipolysis, adipogenesis and adipose tissue insulin resistance. The aims of this review are to investigate the subtle balances that underlie lipolytic, lipogenic and oxidative pathways, to evaluate critical points and the complexities of these processes and to better understand which are the metabolic derangements resulting from their imbalance, such as type 2 diabetes and non alcoholic fatty liver disease.

## 1. Introduction

Fat accumulates in the presence of excessive caloric intake in order to be used as energy source at a later point in time. In presence of either high fat and/or carbohydrate intake, lipogenesis is stimulated and excess fat is stored as triglycerides (also named triacylglycerols, TAG). During fasting excess plasma free fatty acids (FFA), mainly released by the subcutaneous fat, accumulate in non-adipose tissues (e.g., liver, heart, pancreas and muscle) as triglycerides (TG), and can promote cell dysfunction and death [1]. This phenomenon has different effects dependent on the organ where fat accumulates [2]. Hence excess TG in the liver results in hepatic steatosis, fibrosis and non-alcoholic steatohepatitis (NASH) [3,4]; fat in the pancreas is associated with impaired insulin secretion, β-cell dysfunction and apoptosis [5,6]; excess intramyocardial fat leads to cardiomyopathy, coronary heart disease and sudden death [7,8]; in the skeletal muscles, intramyocellular TGs are associated with insulin resistance and impaired glucose uptake [1,9]. Alterations in lipogenesis and lipolysis are both causes and consequences of insulin resistance [1,7,10,11], since the imbalance in lipid metabolism is the primary cause of lipotoxicity.

In this manuscript we review the mechanisms that regulate lipid synthesis, lipolysis and oxidation in order to understand which are the “*primum movens*” of metabolic disorders, such as obesity, diabetes, non alcoholic fatty liver disease (NAFLD) and cardiovascular disease (CVD), including endothelial dysfunction, atherosclerosis and coronary heart disease (CHD).

## 2. Lipogenesis

TG synthesis is a crucial and strictly regulated process that occurs principally in the adipose tissue, but also in the liver, muscle, heart and pancreas. This pathway is used to maintain and control energy homeostasis by a continuous communication between oxidative tissues and peripheral organs, in particular adipose tissue.

**Figure 1:**
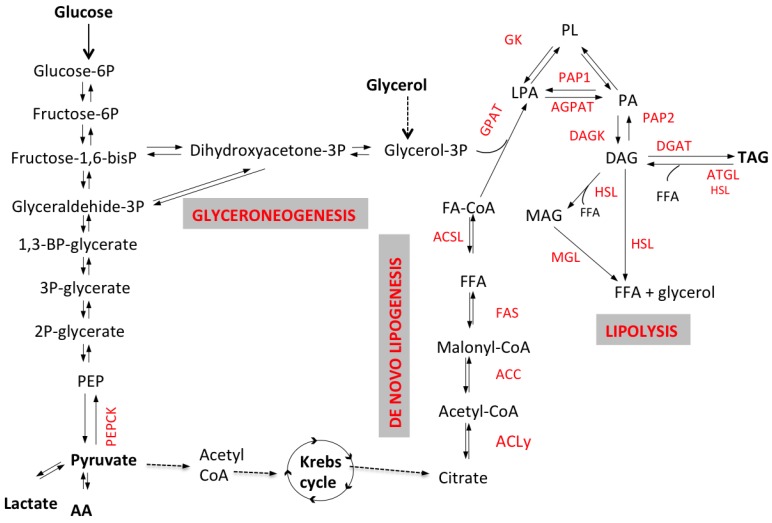
Schematic representation of lipolytic and lipogenic pathways. Triacyglycerol (TAG) synthesis requires the activation of free fatty acids (FFA) into Acyl-CoA by enzyme acyl-CoA synthetase. FFA-CoA and G3P are transformed via acylation, by glycerol-3-phosphate acyltransferase (GPAT) and acylCoA acylglycerol-3-phosphate acyltransferases (AGPAT), to phosphatidic acid (PA); then, after a dephosphorylation by phosphohydrolase (PAP2), diacylglycerols (DAG) are formed. Diacylglycerol acyltransferase (DGAT) catalyzes the conversion of DAG into TAG. In the adipocyte, G3P might come either from glycolysis or from non-carbohydrate substrates via the enzyme phosphoenolpyruvate carboxykinase (PEPCK), through a process named glyceroneogenesis. In the liver G3P can also be synthesized from plasma glycerol. *De novo* fatty acids synthesis (also referred to as *de novo* lipogenesis or DNL) occurs in the cytoplasm of various cells (e.g., adipocytes and hepatocytes) where citric acid is converted to acetyl-CoA by ATP-citrate lyase (ACL) and subsequently to malonyl-CoA by acetyl-CoA carboxylase (ACC). DNL occurs mainly in the liver, but it might occur in adipose tissue as well, although with low rates. This process requires the two enzymes ATP-citrate lyase (ACL), acetyl-CoA carboxylase (ACC) and the multi-enzymatic complex fatty acid synthase (FAS). G3P can be synthesized directly from non-carbohydrate substrates such as pyruvate, lactate or amino acids in oxaloacetate, that is converted to G3P either directly from phoenolpyruvate (PEP), via the key enzyme phosphoenolpyruvate carboxykinase (PEPCK), or through synthesis of dihydroxyacetone (DHA). TAG catabolism (*i.e.*, lipolysis) involves several lipases, adipose triglyceride lipase (ATGL), hormone-sensitive lipase (HSL) and monoacylglycerol lipase (MGL) and produces the release of three free fatty acids (FFA) and one glycerol molecule.

The process of fatty acid esterification into TAG involves the activation of FFA into Acyl-CoA through the formation of monacylglycerol (MAG) and diacyglycerol (DAG) by reacting with glycerol-3-phosphate (G3P) (Figure 1). Several hormones control lipogenesis including insulin that stimulates lipid synthesis and adipogenesis, while glucagon and catecholamines promote acetyl-CoA carboxylase (ACC) phosphorylation and inhibit fatty acids (FA) synthesis. Sources of G3P and Acyl-CoA are plasma glycerol and FFA, but these substrates may also be synthesized *de novo.* The contribution of *glyceroneogenesis* and *de novo lipogenesis* to hepatic TG synthesis is significant, particularly in conditions of insulin resistance, and might be a target for drug intervention. Below we discuss the different pathways involved in lipogenesis and how they are altered in metabolic diseases, particularly NAFLD and type 2 diabetes (T2DM).

### 2.1. Glycerol-3-Phosphate (G3P) Synthesis and Glyceroneogenesis

The first step of FFA esterification is the reaction with G3P. In adipose tissue the main source of G3P is glucose via glycolysis, since the activity of glycerokinase (GK), the enzyme that transforms glycerol into G3P, is low. This process is stimulated by insulin that promotes the uptake of glucose into the cell but also the transformation of dihydroxyacetone-3P (DHAP) into G3P by glycerophosphate dehydrogenase (Figure 1) and finally the reaction with FFA to synthesize TAG.

G3P can also be synthesized from non-carbohydrate substrates such as pyruvate, lactate or amino acids through *glyceroneogenesis* that plays a significant role both in adipose tissue and the liver [12] (Figure 1). Since the liver expresses GK, it has been thought that during lipogenesis the main substrate for TG synthesis was plasma glycerol. Studies analyzing plasma very low density lipoprotein (VLDL)-TG composition after ingestion of deuterated water (used as precursor of glyceroneogenesis) have shown that, during the synthesis of TAG, the liver utilizes mainly glycerol derived from glyceroneogenesis (over 54%), while the rest of the glycerol derives either from plasma glycerol (30%) or from plasma glucose through glycolysis (12%) [13]. Thus, glyceroneogenesis is an important pathway in TAG synthesis, while it is likely that the liver utilizes circulating glycerol as gluconeogenic substrate rather than using it for TAG synthesis. Hepatic gluconeogenesis and glyceroneogenesis have the synthesis of glyceraldehyde-3P (Figure 1) in common. We have shown that FFA and visceral fat accumulation are both associated with increased gluconeogenesis, and it is likely that glyceroneogenesis is also increased thus explaining the positive correlation between hepatic and visceral fat [14]. Thiazolidinediones decrease hepatic fat and gluconeogenesis [15,16,17] and promote adipose tissue glyceroneogenesis and TAG re-esterification [18]. The activation of these pathways explains the increase in subcutaneous fat and the decrease in hepatic and visceral fat observed after thiazolidinediones treatment [15,16]. However, data on this topic are still limited and more studies are needed.

### 2.2. De Novo Lipogenesis

TGs are synthesized either from circulating FFA derived from the diet, peripheral lipolysis or *de novo* lipogenesis (DNL). DNL occurs primarily in the liver and mostly after a high-carbohydrate meal when only part of the carbohydrates are stored as hepatic glycogen while the excess is converted to fatty acids and TAG [19]. During glycolysis citric acid is converted to acetyl-CoA, malonyl-CoA and palmitate, the first fatty acid synthesized (Figure 1). Other fatty acids are then produced through different mechanisms, e.g., stearic acid by elongation of palmitic acid, palmitoleic acid and oleic acid by desaturation of palmitic and stearic acid respectively.

**Figure 2:**
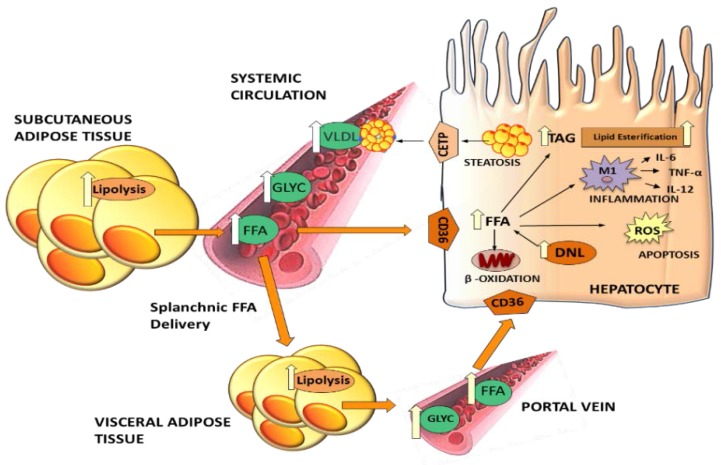
Effects of increased lipolysis on liver dysfunction. Excess lipolysis results in high free fatty acid (FFA) flux into the liver, where FFAs cause steatosis and exert lipotoxic effects. Triglycerides (TAG) synthetized in the liver are secreted into the plasma circulation as very low density lipoproteins (VLDL) causing dyslipidemia. Visceral fat has a preferential role in hepatic fat accumulation since released FFA reach the liver via the portal vein. Also increased hepatic *de novo* lipogenesis (DNL), inflammation and oxidative stress contribute to liver damage and hepatocyte dysfunction.

Contribution of DNL to the TAG pool is crucial in the balance between lipolysis and lipogenesis; however, the contribution of DNL to TAG synthesis is still unknown, mainly because it is difficult to measure it *in vivo* in humans. Published data indicate that increased DNL rates contribute to excess hepatic TAG synthesis and deposition, causing NAFLD [20]. The rate of *de novo* synthesis of palmitate in humans has been measured using the deuterated water technique [20]. DNL contributes to 10%–35% of the total VLDL-TG pool [21], being higher in overweight compared to lean subjects and in general in insulin resistant states, including NAFLD [20,22,23,24]. Moreover, higher rates of DNL are associated with high postprandial glucose and insulin concentrations [21,25], inflammation, oxidative stress and macrophage infiltration [26] (Figure 2).

Possible mechanisms that explain increased hepatic DNL are the activation of the transcription factors Sterol Response Element Binding Protein 1c (SREBP-1c) and Carbohydrate Response Element Binding Protein (ChREBP). SREBP-1c and ChREBP regulate the expression of the key lipogenic genes acetyl-CoA carboxylase (ACC), fatty acid synthetase (FAS), acetyl-CoA synthetase (ACSS) and ATP-citrate lyase (ACL) [27,28]. SREBP-1c seems to be the predominant regulator of DNL in the liver but not in adipose tissue. In contrast, ChREBP regulates DNL in adipocytes where, unlike in liver cells, it has beneficial metabolic effects since it improves insulin sensitivity, enhances glucose transporter-2 (GLUT2) receptor expression and glucose uptake [29]. Glucose stimulates ChREBP and Liver X Receptor α (LXRα) expression and gene transcription of ACL, FAS, stearoyl-CoA desaturase-1 (SCD-1) [30]. Insulin stimulates lipogenesis through the SREBP-1c expression [31,32] and lack of its activation has been found to be associated with an increase in insulin induced gene (INSIG-1) mRNA and proteins [33].

Adipose tissue DNL is extremely low, both in lean and obese subjects [34], but it can be involved in the dysregulation of metabolic functions of adipose tissue. In the adipose tissue of morbid obese subjects undergoing bariatric surgery, a low expression of lipogenic genes (*i.e.*, ACC, ACSS and ACL) has been associated with a better outcome and improvement of anthropometric variables after surgery [35].

It is becoming evident that DNL is a possible target for metabolic diseases including NAFLD. Drugs like pioglitazone, a peroxisome proliferator-activated receptor gamma (PPARγ) agonist, and liraglutide, a glucagon like peptide 1 receptor agonist have been shown to reduce liver triglyceride and hepatic steatosis also through reduction of DNL [36,37]. 

### 2.3. Hepatic TG Secretion as Very Low Density Lipoproteins (VLDL)

Hepatic TGs need to be incorporated into VLDL to be secreted in the systemic circulation; alternatively, they are stored in hepatocytes as lipid droplets (Figure 2). Increased hepatic secretion and impaired clearance of VLDL are associated with high plasma concentrations of TG and low density lipoproteins (LDL) and with decreased concentrations of high density lipoprotein (HDL). High plasma TG and LDL and low HDL are well established risk markers of metabolic syndrome, T2DM [38] and CVD, like the development of coronary heart disease and cardiomyopathy [39]. The enzyme that catalyzes TG synthesis is diacylglycerol acyltransferase (DGAT) (Figure 1) and it exists in mammals in two forms, DGAT1 and DGAT2. DGAT1 is expressed ubiquitously, but mainly in the small intestine, muscle and mammary glands, with low levels found in the liver and adipose tissue; DGAT2 is primarily expressed in the liver and adipose tissue [40]. Although both enzymes catalyze similar reactions and esterify diacylglycerol (DAG) into triacylglycerol, their predominant location influences their metabolic effects. Hence DGAT1 is involved in intestinal lipid absorption and chylomicron formation, while DGAT2 is involved in the synthesis of hepatic TG [40,41]. An impairment in DGAT2 activity results in an increase in hepatic DAG accumulation (Figure 1), making the hepatocytes more susceptible to injury by oxidative stress and inflammatory processes, and suggesting a possible contribution of DAG to the development of NAFLD and progression from simple steatosis to NASH [42]. In subjects with metabolic NAFLD, VLDL secretion is often normal or upregulated indicating that this is not the primary mechanism for the development of liver steatosis [43,44,45]. Indeed it seems that VLDL secretion reaches a plateau indicating a sort of saturation [43]. However, subjects with TM6SF2 mutation are more prone to develop NAFLD/NASH due to a genetic defect in VLDL secretion [46], thus they have reduced plasma concentrations of TG and lipoproteins, despite fatty liver, but they are protected against cardiovascular diseases [47].

## 3. Lipolysis

Lipolysis is a catabolic pathway that promotes mobilization of metabolic fuel from adipose to peripheral tissues in response to appropriate energy demands**.** Lipolysis occurs mainly in the adipose tissue. The major determinant of total FFA release is total fat, while gender is not as important. Since fat accumulates mainly in subcutaneous adipose tissue (SAT), this is the main contributor to plasma FFA [48]. The amount of visceral adipose tissue (VAT) is small compared to total SAT, although it may reach more than 38% of the total fat [5], thus its contribution to systemic FFA is minimal.

Lipolysis involves the hydrolysis of TAG that results in the release of fatty acids (FA) and glycerol into the circulation (Figure 1 and Figure 2). TAG hydrolysis requires different steps through the action of lipases (Figure 1). Several lipases have been discovered in the last 20 years. The first step, TAG hydrolysis into DAG, is obtained by adipose triglyceride lipase (ATGL) and results in the release of one fatty acid (Figure 1). Subsequently, DAG are converted by the enzyme monoacyglycerol lipase (MGL) into monoacylglycerols (MAG) with the release of one FFA or are completely hydrolyzed by hormone-sensitive lipase (HSL) with the release of two FFAs and one glycerol.

Adipose triglyceride lipase. ATGL is a member of the patatin-like phospholipase family, also named PNPLA2. ATGL is present in several cell types and is localized on lipid droplets’ surface and in the cytosol. It is activated by fasting, glucocorticoids and peroxisome proliferator-activated receptor (PPAR) agonists and exerts its action preferentially in adipose tissue and in presence of a co-activator protein named comparative gene identification-58 (CGI-58) [49,50]. ATGL is also present in oxidative tissues such as the liver, muscle and heart but here it explicates its action in a different way [51,52,53]. It has been hypothesized that hepatic ATGL might be involved in partitioning and routing TG, either promoting FFA release and oxidation or synthesis of VLDL [54]. Overexpression and improvement of hepatic ATGL activity is associated with increased TG turnover and FFA oxidation while hepatic ATGL deficiency is associated with steatosis [54,55]. However, the role of ATGL is still controversial. Although ATGL knockout mice developed hepatic steatosis and had altered levels of hepatic enzymes ALT/AST they had low inflammation compared to wild type mice indicating a possible protective role of the lack of hepatic ATGL against progression to NASH [53,55,56]. Muscle ATGL also has a crucial role in the activation of lipolysis and the prevention of intramuscular lipid accumulation. Exercise and physical activity increase ATGL expression in the muscle, promoting fatty acid utilization and oxidation [50]. A recent study has shown that in the heart, mice overexpressing ATGL are protected against cardiac steatosis and development of cardiac dysfunction [57]. These results suggest a different action of ATGL in non-adipose tissues, but more investigations are necessary to better understand the real functions of this lipase.

Monoacyglycerol lipase (MGL). Another important lipase is MGL that catalyzes the last step of lipolysis, *i.e.*, the transformation of MAG, derived either from TG extracellular hydrolysis (via lipoprotein lipase, LPL), or TG intracellular hydrolysis into one FFA and one glycerol (Figure 1). MGL is ubiquitous in tissues (*i.e.*, it is present in adipose tissue, muscle, liver and heart) but the highest expression is shown in adipose tissue. MGL acts on MAG coming from different sources, intracellular and extracellular TG hydrolysis but also phospholipid hydrolysis.

Hormone-sensitive lipase. HSL is an intracellular neutral lipase capable of hydrolyzing several lipid esters including TG, DAG, MAG, and cholesteryl esters, as well as other lipid and water soluble substrates [58]. However, the main activity of HSL is to hydrolyze DAG into MAG or completely with release of FFAs (Figure 1). HSL is activated mainly by β-adrenergic stimulus and inactivated by insulin, but it needs to be phosphorylated and translocated into lipid droplets to explicate its activity. Early studies in the 1960s concluded that HSL was the rate-limiting enzyme for TG hydrolysis but subsequent studies in knockout HSL mice contributed to elucidate the role of this enzyme. HSL-deficient mice were able to efficiently hydrolyze TG [59], showed no increased TG accumulation in either adipose tissue or liver but had an increased accumulation of DAG that interfered with normal cell metabolism and function [60].

## 4. Insulin Resistance and Lipolysis

The secretions of several hormones, *i.e.*, catecholamines, glucagon and insulin, are altered in insulin resistant states. These hormones are important since they control lipolysis through direct or indirect pathways. Catecholamines exert the most potent action to promote this catabolic pathway and stimulate lipolysis [61,62]. Glucagon also acts as a lipolytic hormone that stimulates breakdown of triglycerides from lipid droplets [63]. Insulin exerts the opposite action, promoting adipogenesis and inhibiting lipolysis [64]. Higher levels of insulin in the blood are observed in insulin resistant subjects since there is a greater demand of insulin secretion by the beta cells to facilitate peripheral glucose uptake [64].

**Figure 3:**
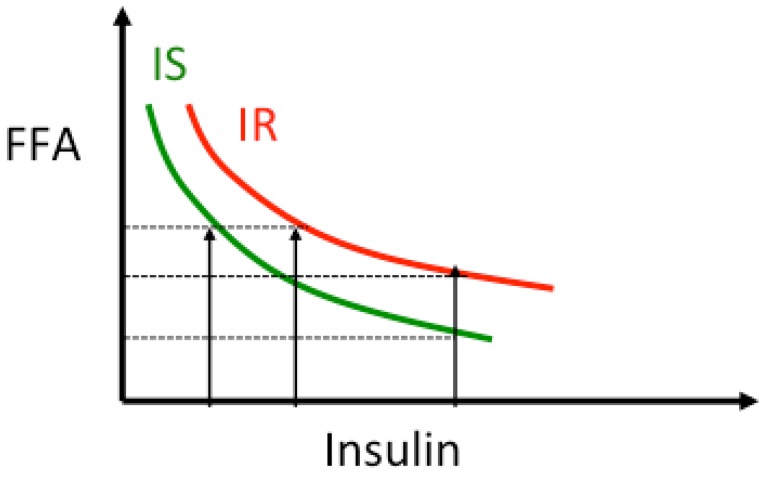
Relationship insulin-lipolysis. As insulin concentration increases, lipolysis, and thus plasma free fatty acids (FFA) concentration, is suppressed following a non-linear curve [65,69,70]. In presence of insulin resistance the curve is shifted to the right indicating that for the same insulin levels lipolysis is less suppressed and circulating FFA levels are higher. The product FFA × Insulin is used as an index of adipose tissue-insulin resistance.

Insulin resistance is often present at the levels of all organs, muscle, liver, heart and adipose tissue, where insulin promotes FA esterification and synthesis of TAG. In addition, insulin suppresses adipose tissue lipolysis and the release of free fatty acid (FFA). The dose-response curve of FFA *vs.* insulin follows a hyperbolic curve [65]. As subjects become more insulin resistant at the level of adipose tissue the curve shifts to the right indicating that for the same insulin levels plasma FFA are higher (Figure 3). In this condition elevated plasma FFA reduce basal and insulin-stimulated muscle glucose uptake by inhibiting insulin signaling [9]. FFA decrease muscle ATP synthesis [66] and nitric oxide production [67], and impair insulin-stimulated activation of phosphoinositol-3 kinase (PI3K), pyruvate dehydrogenase kinase-isozyme 1 (PDK1), RAC-alpha serine/threonine-protein kinase (also known as proto-oncogene c-AKT), and endothelial nitric oxide synthase (eNOS) [67]. Moreover, high FFA are associated with increased cellular levels of diacylglycerol (DAG), the first step of TAG synthesis (Figure 1). Other lipid metabolites are increased in insulin resistant states, e.g., ceramide, and long-chain fatty acyl-coenzyme A (CoA), and activate transcription factors such as nuclear factor-κB (NF-κB) and inflammatory processes [68].

Adipose tissue insulin resistance index (Adipo-IR) has been developed to evaluate the degree of antilipolytic effect of insulin. Considering the hyperbolic relationship between FFA and insulin, Adipo-IR is calculated as the product of FFA × insulin [14,65] or as the product of rate of lipolysis × insulin [69,70]. Often FFA concentrations are not increased in the fasting state in insulin resistant subjects [63], but because of higher insulin concentrations the dose response curve is shifted to the right (as shown in Figure 3) [63,64]. On the other hand, suppression of lipolysis at higher insulin levels, e.g., after a glucose load, a meal test or during insulin infusion, is greater in insulin sensitive than insulin resistant subjects (Figure 3). Similar results were observed in non obese patients with NAFLD compared to matched controls [69]. 

In subjects with insulin resistance, e.g., obese, type 2 diabetes, NAFLD *etc.*, the Adipo-IR has been found to be increased proportionally to visceral and hepatic fat [3]. Patients with abdominal and ectopic fat accumulation are “metabolically abnormal” compared to subjects with similar total body fat [71], they are more resistant to the antilipolytic effect of insulin with increased fasting lipolysis, but similar FFA concentrations, and impaired suppression of palmitate release during insulin infusion [71].

Excess FFA release not only causes peripheral insulin resistance [68], but also increases insulin secretion and impairs beta cell function [64,72]. Kashyap *et al.* have shown that chronic (48 h) intravenous infusion of an intralipid emulsion of essential saturated and unsaturated fatty acids plus heparin induces peripheral insulin resistance and stimulates insulin secretion in subjects without a family history of diabetes (FHD) while it markedly impairs insulin secretion in subjects with FHD [72]. The same type of response was observed in human islets incubated with fatty acids [73].

## 5. Dysfunctional Adipose Tissue: Accumulation and Remodeling

Adipose tissue expansion is a dynamic process that occurs in obesity, but is not always associated with pathological processes [74]. Subcutaneous fat is the main site of fat accumulation but visceral adipocytes are more resistant to the antilipolytic effect of insulin and catecholamines [75,76]. Visceral adipocytes are more lipolytic than subcutaneous adipocytes when incubated with different concentrations of norepinephrine, proportionally to the hepatic fat content [75]. Visceral fat (VF), more than subcutaneous fat, is associated with metabolic abnormalities including insulin resistance and lipotoxicity through the increased release of cytokines and decreased release of adiponectin. For example, visceral adipocyte diameter is higher in patients with more severe NAFLD and was found increased with serum levels of ALT and C- reactive protein [45]. Moreover, VF releases FFA directly into the portal vein and they are, therefore, cleared mainly by the liver [3,5]. Subjects with visceral fat have higher postprandial FFA and are at a higher risk of NAFLD and hepatic insulin resistance [14] (Figure 2).

**Figure 4:**
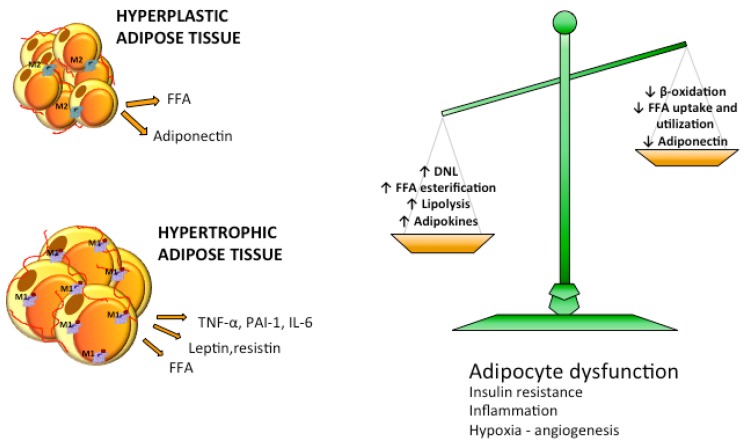
Imbalance in lipid metabolism causes increased efflux of FFA to adipose tissue. Reduced free fatty acids (FFA) utilization and β-oxidation and increased lipogenic and lipolytic pathways lead to overflow of FFA in the circulation. Adipose tissue activates adipogenesis and increases the number of adipocytes becoming hyperplastic or enlarges adipocyte size becoming hypertrophic. Hyperplastic adipose tissue is normally metabolically healthy while hypertrophic adipose tissue is characterized by dysfunctional adipocytes, insulin resistance, hypoxia and inflammation.

During adipose tissue expansion adipocytes become either hyperplastic, when their number increases through adipogenesis, or hypertrophic, when their size increases via lipogenesis [77] (Figure 4). Adipocytes act both as energy storage and as endocrine organ, being able to produce and release hormones, such as leptin, that is involved in the regulation of appetite; adiponectin, implicated in fatty acid oxidation and insulin action; cytokines like IL6 and tumor necrosis factor-α (TNF-α) that are involved in the regulation of lipolysis and can activate the complement system and vascular homeostasis [78,79,80] (Figure 4). Adipocyte cell size correlates positively with secretion of proinflammatory adipocytokines, e.g., leptin, inteleukin 6 and 8 (IL-6, IL-8), and monocyte chemoattractant protein-1 (MCP-1), as shown by data from cultured adipocytes [81]. In humans, visceral adipocyte size correlates directly with leptin [45] and inversely with adiponectin [82]. Adipose tissue expansion is regulated by storage-related genes like DGAT2, SREBP1c and cell death activator (CIDEA). A hypercaloric diet upregulates lipogenic genes in the adipose tissue [71]. Interestingly it has been shown that when the regulation of these genes in subcutaneous tissue is defective, the subjects tend to accumulate more visceral and ectopic fat [83]. 

Some obese subjects preserve insulin sensitivity and lipid homeostasis and they are called “metabolically healthy obese” or MHO [71,84]. It has been demonstrated that adipose tissue morphology, more than the total amount of fat, plays an important role in the worsening of glucose and lipid metabolism [79]. Thus, in “metabolically healthy obese” adipocytes tend to be smaller than in obese insulin resistant subjects [85,86], where adipose tissue hypertrophy is accompanied by hypoxia, overproduction of pro-inflammatory cytokines, cellular fibrosis and macrophage infiltration [84,87,88] (Figure 4). Non-obese individuals at risk of T2DM are more prone to develop an obese phenotype with dysregulated adipose tissue, hypertrophic enlargement of adipocytes and reduced circulating adiponectin levels and glucose transporter-4 (GLUT4) expression for glucose uptake [89]. A recent study performed in 29 young healthy men has proposed that adipocyte size is predictive of the response to excess energy intake and could play a role in insulin resistance and inflammatory answer of adipose tissue [86]. Unexpectedly they showed that lean subjects with smaller fat cells responded to 8 weeks excess energy and lipid intake with a rapidly and not protective remodeling, developing insulin resistance, expansion of subcutaneous fat and up regulation of inflammatory markers. In contrast participants with larger subcutaneous adipocytes developed less insulin resistance and ectopic/visceral fat accumulation, probably due to a reduced expandability of these cells [86].

Adipocytes are among the most insulin-sensitive cells. When adipocytes become dysfunctional they become resistant to the anti-lipolytic effect of insulin resulting in a huge increase in the release of FFA and adipokines such as TNF-α and monocyte chemoattractant protein-1 (MCP-1) that play a key role in the development and maintenance of insulin resistance status. TNF-α induces insulin resistance in adipose tissue by altering the normal insulin signaling pathway, stimulating adipocytes lipolysis, decreasing insulin receptor substrate-1 (IRS-1) activity and its substrate phosphorylation and decreasing glucose transporter GLUT4 synthesis and membrane translocation. TNF-α leads to insulin resistance also in non-adipose tissues such as liver and muscle and promote FA mobilization from adipose tissue to oxidative tissues [90]. MCP-1 contributes to microphage infiltration in adipose tissue, insulin resistance and NAFLD [91] (Figure 4). Metabolomic analysis of subcutaneous adipose tissue found that several aminoacids, phosphocholines, ceramides and sphingolipids were increased in insulin resistant *vs.* insulin sensitive obese subjects and correlated with Adipo-IR [92].

## 6. Lipid Oxidation

**Figure 5:**
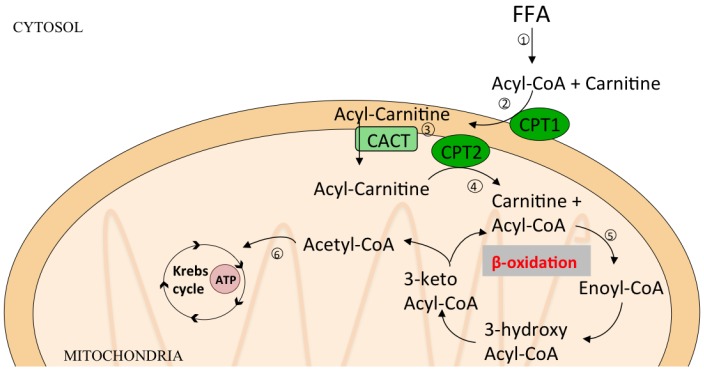
Pathways of β-oxidation. β-oxidation is the catabolic pathway that occurs in mitochondria and produces energy from TG hydrolysis. (1) FFA are transformed to Acyl-CoA in cytosol; (2) protein Carnitine Palmitoyl Transferase-1 (CPT1) catalizes the transfer of the acyl group of a long-chain fatty acyl-CoA to carnitine to form acylcarnitines (mainly Palmitoylcarnitine); (3) Carnitine Acyltranferase (CACT) transfers acylcarnitine across outer mitochondrial membrane; (4) Carnitine Palmitoyl Transferase-2 (CPT2) reconverts acylcarnitine in acylCoA and carnitine; (5) Acyl-CoA enters in β-oxidation cycle and is degraded in several Acetyl-CoA molecules; (6) Acetyl-CoA enters in Krebs cycle to produce energy as Adenosine Triphosphate (ATP).

The most important catabolic pathway for TAG and FA degradation is β-oxidation that occurs in mitochondria and produces the energy for homeostasis of cells and tissues (Figure 5**)**. The oxidation of fatty acids occurs in particular during fasting state and carbohydrate starvation. In liver mitochondria, the acetyl-CoA produced during β-oxidation is converted to ketone bodies, *i.e.*, acetoacetate, beta-hydroxybutyrate (BOH), and acetone. Ketone bodies are released and then taken up by other tissues such as the brain, muscle or heart where they are converted back to acetyl-CoA to serve as an energy source. Patients with fatty liver not only have increased VLDL-TG synthesis [43,44], but also increased β-oxidation and release of BOH [69]. However, obesity is also associated with increased levels of β-oxidation by the muscles and heart due to elevated circulating concentrations of FFAs that activate PPAR-α.

Glucose and hormones like insulin, glucagon and catecholamines that control lipolysis and lipogenesis modulate substrate availability for β-oxidation. Accelerated glucose metabolism could inhibit β-oxidation due to increased production of pyruvate that is transformed to malonyl-CoA, reducing the fatty acid catabolic pathway [93]. Also excessive FFA can impair mitochondrial function leading to abnormal FA oxidation. In this condition, the mitochondria tend to oxidize more glucose than lipids, even in resting condition. During a stress condition, when there is an increased energy demand, the cell is unable to switch from FA to carbohydrate oxidation. This determines the depletion of Krebs cycle intermediates and accumulation of ACC, thus contributing to insulin resistance (Figure 5).

## 7. Saturated or Unsaturated Fat?

Although unsaturated fatty acids were thought to be more susceptible to oxidative stress, recent work has instead demonstrated that a higher unsaturated/saturated fat ratio is protective against the development of metabolic diseases.

FFA plasma composition is determinant in maintenance of homeostasis. For example, palmitate, compared to other fatty acids, is more “toxic” [94]. Palmitate, but not oleate, impairs hepatic insulin signaling and induces apoptosis in hepatocyte cell lines and also impairs beta cell response. Oleate on the other hand seems to have a “protective” role since the coincubation of the two fatty acids reduces the “toxic” effect of palmitate [94]. This clearly indicates different metabolic signaling of single fatty acids and that different lipid bioactive species could shift the balance towards an adverse metabolic profile. Dietary polyunsaturated fatty acids (PUFA) and conjugated-linoleic acids (CLA) are lipid species that have been shown to have beneficial effects in maintaining lipid homeostasis, promoting loss of adiposity via increasing lipolysis and fatty acid oxidation and inhibiting lipogenesis [95]. These classes of FFA also exhibit anti-inflammatory and anti-oxidative properties via PPAR activation and reduced production of pro-inflammatory cytokines [96,97]. On the contrary saturated fatty acids (SFA) enhance production of reactive oxygen species and proinflammatory cytokines. SFA, in particular palmitic acid, activate mitochondrial depolarization, lead to apoptosis and suppress autophagy and lipid droplet production, which are both protective mechanisms to prevent lipotoxicity [98]. Moreover, SFA are precursors of ceramides that are bad substrates for the synthesis of cardiolipin, an important protein in the mitochondrial membrane. Impaired synthesis of cardiolipin results in increased membrane permeability and release of cytochrome C in the cytosol, causing also in that case apoptosis [99]. Thus, PUFA/SFA ratio has been used as a plasma biomarker of favorable lipid profile.

Animal studies have shown that an increased plasma PUFA/SFA ratio is associated with a favorable serum lipid profile and activation of hepatic enzymes involved in antioxidative pathways [100]. In hamsters, a diet with a high PUFA/SFA ratio prevented fat accumulation in white adipose tissue, increased expression of hepatic lipolytic enzymes, enhanced fatty acid β-oxidation and decreased hepatic SREBP-1c mRNA expression and plasma insulin levels [101]. These data were confirmed in a small group of subjects where an increase in PUFA *versus* SFA dietary intake was associated with reduced abdominal subcutaneous fat, in particular in obese subjects [102].

Plasmatic levels of free fatty acids are considered important parameters of lipolysis, since they reflect fat mobilization from adipose tissue to the circulation in response to energy demand. However, FFA composition and specific ratios are potential biomarkers in chronic metabolic diseases. The palmitate/linoleate ratio (16:0/18:2n6) is considered an index of DNL because it is a ratio of the first and main product of DNL, palmitate, and an essential fatty acid, linoleate, introduced by diet [103,104].

The ratios of palmitoleate/palmitate (16:1n7/16:0) and oleate/stearate (18:1n7/18:0) reflect enzyme activity of stearoyl-CoA desaturase (SCD-1), which add an unsaturation bond to fatty acid precursors palmitate or stearate [105,106,107,108,109,110]. Since SCD-1 activity is referred as the last stage of DNL, increase in either palmitoleate/palmitate (16:1n7/16:0) and/or oleate/stearate (18:1n7/18:0) ratios, especially in fasting state, were associated with an adverse metabolic profile, *i.e.*, visceral fat accumulation, insulin resistance, and increased fasting and post-prandial plasma TG concentrations [104,109].

## 8. Lipotoxicity: Causes and Consequences

**Figure 6:**
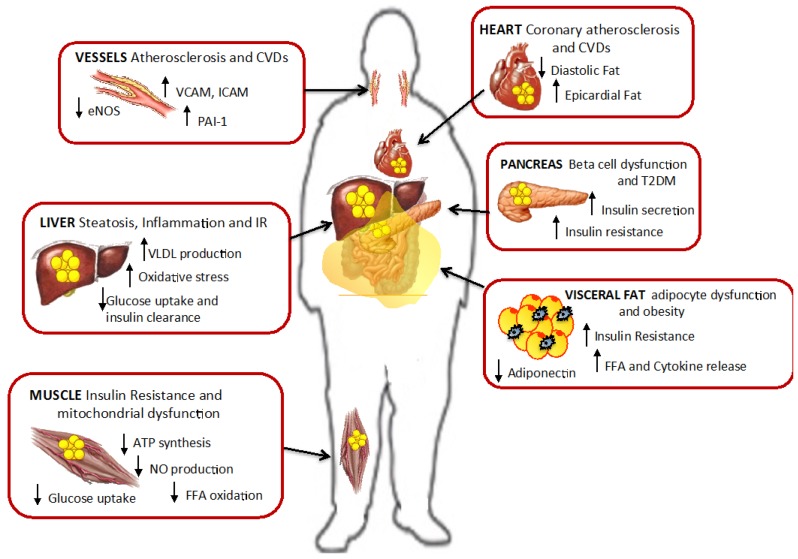
Ectopic fat accumulation and effect of lipotoxicity. Fat accumulation in non-adipose tissues promotes cell dysfunction, insulin resistance and inflammation in liver, muscle, pancreas and visceral fat. Also in vessels and heart lipotoxicity leads to increased risk for cardiovascular diseases and atherosclerosis. Modified from Gaggini M. *et al.* [2].

The imbalance of DNL, lipolysis and β-oxidation results in excess FFA released into the circulation that only in part are taken up by the adipose tissue and the rest by other tissues like the liver, muscle, heart and pancreas [2,111] (Figure 6). In presence of insulin resistance, adipose tissue capacity to metabolize these lipids is limited, so excess lipids also accumulate as ectopic fat and promote lipotoxicity [2]. Lipotoxicity triggers negative effects on multiple cellular processes including impaired insulin signaling [67,112], oxidative stress [113,114], alterations in local renin-angiotensin system [115], enhanced adrenergic sensitivity of vascular smooth muscle cells [116], and mitochondrial dysfunction [111].

### 8.1. The Role of FFA in Lipotoxicity

Several studies have shown that elevation of plasma FFA not only stimulates fat accumulation but also induces cellular transformation by altering the expression of several genes associated with energy metabolism and inflammation, oxidative stress and cell apoptosis.

Elevated plasma FFAs promote peripheral and hepatic insulin resistance, reduce basal and insulin-stimulated glucose uptake in muscle by inhibiting insulin signaling [9]. In the skeletal muscle, fat accumulation reduces translocation of GLUT4 to the plasma membrane in response to insulin stimulation leading to the development of insulin resistance and T2DM [117]. Intramyocellular lipid content causes mitochondrial dysfunction and impaired glucose metabolism correlating with the severity of insulin resistance [118,119]. High FAs decrease muscle ATP synthesis [66], nitric oxide production [112], and impair insulin-stimulated activation of several genes, such as phosphoinositol-3 kinase (PI3K), pyruvate dehydrogenase kinase, isozyme 1, RAC-alpha serine/threonine-protein kinase (also known as proto-oncogene c-Akt), and endothelial nitric oxide synthase (eNOS) [112]. High plasma concentrations of FFAs are also associated with increased markers of endothelial activation, e.g., vascular cell adhesion molecule (VCAM) and intercellular adhesion molecule (ICAM), and increased markers of inflammation, such as plasminogen activator inhibitor-1 (PAI-1) and MCP-1, suggesting an increased risk for atherosclerotic cardiovascular disease [95].

The FFA lipotoxic effect is not limited to the muscle but also extends to other oxidative tissues such as the liver, heart and pancreas (Figure 6). Elevated FFAs stimulate hepatic TG synthesis and production of VLDL [43,44], hepatic insulin resistance, inflammation and fibrosis with the development of fatty liver disease and steatohepatitis [14,120]. Several studies have found a strong association between metabolic syndrome and NAFLD, liver damage, and hepatocellular carcinoma (HCC) [121,122,123,124]. NAFLD/NASH is the second leading etiology of HCC and is currently the most common cause of chronic liver disease [125]. The risk of cancer is high in NAFLD/NASH even in the absence of cirrhosis [126,127], and NAFLD/NASH is currently the most rapidly growing indication for liver transplantation (LT) in patients with HCC [128]. In particular, derangements in lipid metabolism lead not only to hepatic TG accumulation but also to lipotoxicity, oxidative stress and apoptosis [3,98,122,124,129,130]. Adipose tissue insulin resistance is likely to play a significant role since it is related to increased liver damage [124,131]. The mechanisms might be mediated by an increased synthesis of saturated fatty acids, ceramides, phosphatidylcholines, monoacyl-, diacyl- and triacyl-glycerols (MAG, DAG and TAG) and downregulation of lysophosphocholine (LPC), causing mitochondrial dysfunction, oxidative injury and apoptosis by the elevation of lipid peroxides and free radicals [132,133,134].

Ectopic fat can accumulate in the pancreas and induce β-cells dysfunction and dysregulated insulin secretion that is one of the main causes of the onset of T2DM [64]. *In vitro* studies on human β-cells showed that palmitic acid caused a dose-dependent reduction of glucose-stimulated insulin release and an increased cell death [73]. Development of β-cells lipotoxicity in non-diabetic subjects, but genetically predisposed to develop T2DM, was associated with impaired insulin release and secretion [72] (Figure 6).

Cardiac fat accumulates around the heart as pericardial or extrapericardial fat or as intramyocardial TG. Cardiac fat is associated with cardiomyocyte dysfunction and with the development of cardiac disease [7,135] coronary atherosclerosis and calcification [136,137]. Thus, it has been hypothesized that epicardial fat might be responsible for cardiac lipotoxicity, oxidative stress and insulin resistance [7]. However, lipolysis in the epicardial fat results in FFA release directly into the coronaries. Since FFA are the main cadiac energy substrate, epicardial fat, if not hypertrophic and dysfunctional, might have a non harmful role being an immediate source of energy.

### 8.2. Lipidomics and the Discovery of Harmful Lipids

Recent advances in *omics* technology have allowed the accurate identification of several lipid classes and their composition that are possible predictors of metabolic abnormalities. Among these lipids, increased intracellular concentrations of TG, diacylglycerols (DAG), glycerophosphocholine (GPC), phosphocholines (PC), ceramides and sphingomyelin have been implicated in the development of metabolic diseases including diabetes and non-alcoholic fatty liver disease [99,138,139,140,141,142,143,144,145,146,147].

DAGs are lipid intermediates that in normal conditions are converted to TAG or phospholipids (PL) (Figure 1). Recent studies have hypothesized that DAG might be implicated in the development of insulin resistance, inflammatory signaling and also dysmetabolic diseases [42,148,149]. The increment of intracellular DAG is able to activate protein kinase Cε (PKCε) that has an inhibitory effect on phosphorylation of insulin receptor substrate-2 (IRS2). Consequently DAGs promote the development of hepatic insulin resistance and hyperglycemia mainly through lack of suppression of gluconeogenesis. Initial studies in animal models were confirmed in obese subjects with NAFLD where hepatic DAG accumulation was positively correlated with hepatic insulin resistance [150]. Several analyses of liver tissue (both normal and steatotic livers) in human and murine specimens revealed a dramatic fold change in DAG composition, in particular an increase in DAG containing monounsaturated fatty acids [151].

Ceramides are molecules derived from sphingolipids, and they have been implicated in apoptosis [152]. Increased serum levels of ceramides were associated with insulin resistant states. However, there are more than 200 ceramides, so it is possible that not all lipids, but only some of them, are really implicated in cell damage and inflammation [68]. In particular, recent analysis of lipidomic data focused on the number of double bonds (*i.e.*, degree of desaturation) as a possible way to find lipid biomarkers of disease [149].

## 9. Summary and Conclusions

A complex network of pathways, responding to several endogenous and exogenous stimuli, characterizes lipid metabolism. The alterations in adipogenesis, lipolysis and lipid oxidation are key factors in the development of metabolic disorders such as obesity, diabetes, NAFLD and CVD. Excess lipolysis results in excess release of FFAs into the circulation that are then taken up not only by adipose tissue but also by the liver, muscle, pancreas and/or heart, thus limiting excursions in plasma FFA concentrations and generating a lipotoxic profile in the organs. However, not all obese subjects are insulin resistant and have alterations in lipolysis and lipogenesis, and only those that develop cellular lipotoxicity are at risk of metabolic disorders. 

The recent development of *omics* techniques allowed the discovery of plasma and tissue biomarkers of lipotoxicity. These are molecules that mark altered lipid mechanisms involved in the onset and/or progression of metabolic diseases, such as diacylglycerols (DAG), ceramides and long-chain fatty acids. The exposure to high FFA increases the production of these lipid intermediates and metabolites. These compounds are able to activate transcription factors involved in inflammatory processes and oxidative stress, leading to lipotoxicity. A lot of work still needs to be done and only the multi-*omics* approach, e.g., lipidomics, metabolomics, transcriptomics, genomics and fluxomics, will elucidate pathways that are still unclear and determine which molecules are implicated.

In conclusion, lipids appear to be key players in metabolic derangement, especially when they accumulate as visceral or ectopic fat. In this condition, lipids exert a lipotoxic action, causing cell dysfunction and organ damage. Extensive knowledge of mechanisms involved in lipid metabolism and its control is necessary to identify early biomarkers of cardio-metabolic diseases, new pharmacological strategies and to provide new behavioral lifestyle interventions.

## Abbreviations

ACC (acetyl-CoA carboxylase); ACL (ATP-citrate lyase); Adipo-IR (adipose tissue insulin resistance index); AGPAT (acylCoA acylglycerol-3-phosphate acyltransferases); AKT1 (RAC-alpha serine/threonine-protein kinase); ALT (alanine transaminase); AMPK (AMP- Activate protein kinase); AST (aspartate transaminase); ATGL (adipose triglyceride lipase); ATP (Adenosine Triphosphate); CACT (Carnitine Acyltranferase); CEPT (Cholesteryl ester transfer protein); CGI-58 (gene identification-58); ChREBP (Carbohydrate Response Element Binding Protein); CIDEA (Cell death activator); CLA (conjugated-linoleic acids); CPT1 (Carnitine Palmitoyl Transferase1); DAG (Diacylglycerols); DAGK (diacylglycerol kinase); DGAT (diacylglycerol acyltransferase); DHAP (dihydroxyacetone-3-phosphate); DNL (*De novo* lipogenesis); eNOS (endothelial nitric oxide synthase); FAS (fatty acid synthetase); FFA (free fatty acids); G3P (Glycerol-3-phosphate); GLP-1 (glucagon like peptide 1); GLUT-2 and GLUT-4 (glucose transporter); GPAT (glycerol-3-phosphate acyltransferases); GPC (glycerophosphocholine); HCC (hepatocellular carcinoma); HDL (high density lipoprotein); HSL (hormone-sensitive lipase); ICAM (Intercellular Adhesion Molecule); INSIG-1 (insulin induced gene); IRS-1 and IRS-2 (insulin receptor substrate); LDL (low density lipoprotein); LPC (lysophosphocholine) LXRα (Liver X Receptor α); MAG (Monoacylglycerols); MCP-1 (Monocyte chemoattractant protein-1); MGL (monoacylglycerol lipase); MUFA (monounsaturated fatty acids); NAFLD (Non Alcoholic Fatty Liver Disease); NAFLD (nonalcoholic fatty liver disease); NASH (Non Alcoholic Steatohepatitis); PAI-1 (plasminogen activator inhibitor-1); PAP (phosphohydrolases); PC (phosphocholine); PDK1 (pyruvate dehydrogenase kinase-isozyme 1); PEP (phoenolpyruvate); PEPCK (phosphoenolpyruvate carboxykinase); PI3K (phosphoinositide 3-kinase); PKCε (Protein kinase C isoform ε); PL (phospholipids); PNPLA2 (patatin-like phospholipase); PPAR (peroxisome proliferator-activated receptor); PUFA (polyunsaturated fatty acids); SCD-1 (stearoyl-CoA desaturase-1); SFA (saturated fatty acids); SREBP-1c (Sterol Response Element Binding Protein 1c); T2DM (type 2 diabetes); TAG (Triacylglycerols); TNF-α (tumor necrosis factor-α); VCAM (vascular cell adhesion molecule); VLDL (very low density lipoprotein).
